# Immunoinflammatory markers SIRI and NAR as predictors of respiratory distress syndrome and secondary infections in premature infants

**DOI:** 10.3389/fcimb.2024.1512884

**Published:** 2024-12-13

**Authors:** Xuexin Chen, Yuedong Li, Hongxiang Chen, Weiming Chen

**Affiliations:** ^1^ Department of Neonatology, Meizhou People’s Hospital, Meizhou Academy of Medical Sciences, Meizhou, China; ^2^ Data Center, Meizhou People’s Hospital, Meizhou Academy of Medical Sciences, Meizhou, China

**Keywords:** respiratory distress syndrome, premature infants, respiratory infection, system inflammation response index, neutrophil-to-albumin ratio

## Abstract

**Background:**

Premature infants are at high risk for neonatal respiratory distress syndrome (RDS) and secondary infections. This study aims to investigate the association between immunoinflammatory markers—the systemic immune inflammation index (SII), systemic inflammation response index (SIRI), and neutrophil-to-albumin ratio (NAR)—and the risk of developing RDS in premature infants.

**Methods:**

A total of 2164 premature infants were enrolled in this retrospective study. The clinical records of these neonates (respiratory tract infections, adverse pregnancy history, placental abnormalities, birth weight, Apgar scores, and immunoinflammatory indices) were collected. Comparisons were made between infants with and without RDS. Logistic regression analysis was used to evaluate the relationship between SII, SIRI, NAR and RDS.

**Results:**

Among the cohort, 962 infants developed RDS, while 1202 did not. The RDS group showed higher proportions of mothers with adverse pregnancy history, placental abnormalities, birth weight <2.5 kg, and lower Apgar scores at 1 and 5 minutes (all *p*<0.05). SII, SIRI, and NAR levels were significantly elevated in RDS infants (*p*<0.05). Logistic regression revealed that adverse pregnancy history (OR: 1.390, *p*=0.001), placental abnormalities (OR: 2.499, *p*<0.001), birth weight <2.5 kg (OR: 4.165, *p*<0.001), high SIRI (OR: 1.338, *p*=0.035), and high NAR (OR: 1.639, *p*<0.001) were significant predictors of RDS. Additionally, secondary infections, particularly pneumonia and sepsis, were significantly more common in the RDS group (*p*<0.001).

**Conclusion:**

Adverse pregnancy history, placental abnormalities, low birth weight, elevated SIRI and NAR were associated with increased risk of RDS and secondary infections in premature infants. These findings suggest that SIRI and NAR could serve as useful markers for early identification and management of RDS and its complications in this vulnerable population.

## Introduction

Premature birth is an important challenge in perinatal medicine (Stevenson et al., 2024). The American College of Obstetricians and Gynecologists (ACOG) defines preterm birth as a birth of less than 37 weeks gestation (Di Renzo et al., 2017). In China, preterm birth is defined as delivery from 28 weeks to less than 37 weeks of pregnancy (Cao et al., 2022; Song et al., 2022). In 2012, the Global Prematurity Action Report issued by the World Health Organization (WHO), the first estimate of the world’s preterm birth rate, estimated that about 15 million babies are born prematurely every year, more than 10% of all births (Blencowe et al., 2013). The nationwide incidence of preterm birth was 7.3% of all births and 6.7% of live births in China (Chen et al., 2019a), and there were regional differences (Hu et al., 2024a).

Premature infants are prone to have some complications, such as neonatal entrapment, neonatal respiratory distress syndrome (RDS), infection, necrotizing enterocolitis, brain injury, bronchopulmonary dysplasia, and so on (Cheong et al., 2020; Zhao et al., 2024, 2021; Jensen et al., 2021). Neonatal RDS refers to the progressive exacerbation of respiratory distress caused by lack of pulmonary surfactant after birth (Dargaville et al., 2023; Ramaswamy et al., 2022). It is a hypoxic respiratory failure caused by lung gas exchange disorders and pulmonary mechanical abnormalities caused by intrapulmonary or extrapulmonary causes, resulting in extensive acute inflammatory lung injury (Chen et al., 2014; Ketcham et al., 2020; Saguil and Fargo, 2020). Premature infant RDS is the main cause of neonatal death due to its rapid onset, rapid progression and high disability rate (Guo and Fan, 2021). The pathogenesis of RDS has not been fully explained. The main pathogenesis of RDS is the uncontrolled local inflammatory response of the lung and the damage of lung epithelial cells and endothelial cells mediated by it, which leads to the increase of pulmonary vascular permeability and the decrease of pulmonary surfactant (Huppert et al., 2019). The main pathophysiological changes of RDS are the acute uncontrolled inflammatory response of lung tissue caused by various pathogenic factors inside and outside the lung, resulting in alveolar capillary injury, increased permeability, induced pulmonary edema, and then progressive dyspnea and hypoxemia in newborns (Huppert et al., 2019).

Inflammatory cells in the body include neutrophils, lymphocytes, monocytes, and platelets (Xie et al., 2023; Almăşan and Leucuţa, 2022). Systemic immune inflammation index (SII) and system inflammation response index (SIRI) are important markers of systemic immune inflammation based on neutrophils, lymphocytes, monocytes, and platelets counts, and their links to several diseases are being revealed (Song et al., 2023; Islam and Satici, 2024; Jarmuzek and Kozlowska, 2023). In addition, the inflammatory environment induces neutrophil flow and activation in the lungs and promotes the release of neutrophil extracellular traps (NETs) (Blanch-Ruiz and Ortega-Luna, 2021). NETs are associated with acute respiratory distress syndrome (ARDS) (Blanch-Ruiz and Ortega-Luna, 2021). Neutrophil-to-albumin ratio (NAR) is an important index that comprehensively reflects the level of systemic immunity and nutritional status and has been proven to be closely related to some diseases (Karasu et al., 2023; Zhang et al., 2022). Ufuk Cakir et al. demonstrated that a higher SII level was a predictor for RDS among premature infants (Cakir et al., 2024). There is no reported relationship between SIRI, NAR and neonatal RDS. The purpose of this study was to analyze the predictive value of SII, SIRI and NAR in neonatal RDS risk by retrospective case-control study, and to analyze the risk factors of neonatal RDS by including perinatal-related factors.

## Materials and methods

### Subjects

It was a retrospective study with a total of 2164 premature infants who were hospitalized in Meizhou People’s Hospital, from October 2018 to March 2024. In this study, premature birth is defined as delivery less than 37 weeks according to neonatal gestational age at birth. The diagnostic criteria of the Montreux Criteria for ARDS (De Luca et al., 2017): (1)acute onset from a known or suspected clinical insult; (2)primary current acute respiratory diseases caused by congenital abnormalities were excluded; (3)diffuse, bilateral, and irregular opacities or infiltrates, or complete opacification of the lungs; (4)echocardiography is needed to verify the origin of oedema; and (5)oxygenation deficit.

Inclusion criteria of patients with RDS: (1) neonates met the diagnostic criteria of Montreux Criteria; (2) premature infants with gestational age of less than 37 weeks; (3) clinical data were complete. Premature infants without RDS born in our hospital during the same period were selected as non-RDS group. Inclusion criteria of non-RDS group: (1) premature infants with gestational age of <37 weeks; (2) clinical data were complete. Exclusion criteria of patients with RDS and non-RDS: (1) severe cardiac, liver, and kidney dysfunction at birth; (2) newborns with autoimmune disorders or severe infectious diseases; (3) neonates with congenital genetic diseases. These premature infants included 962 patients with RDS and 1202 newborns without RDS. This study was supported by the Ethics Committee of the Meizhou People’s Hospital.

### Collected and observed indices

Clinical data of the two groups of neonates and their mothers were collected from the medical records system of our hospital, including: (1) data of neonates’ mothers: adverse pregnancy and birth history (spontaneous abortion, embryo discontinuance, fetal malformation or defect, postpartum hemorrhage, and so on), pregnancy conditions (amniotic fluid contamination, nuchal cord, placental abnormality, and mode of delivery); (2) data of neonates: gender of neonates, birth weight, 1-minute Apgar score, 5-minute Apgar score, and indices of immune-nutritional status. The newborns’ venous blood was collected, blood cell analysis was tested by the Sysmex XE-2100 haematology analyzer (Sysmex Corporation, Japan) and serum albumin was measured by Roche automatic biochemical analyzer.

### Data processing and statistical analysis

The indices of immune-nutritional status: SII, SIRI, and NAR were calculated according to the following formula:

SII=platelet×neutrophil/lymphocyte.

SIRI=monocyte×neutrophil/lymphocyte

NAR=neutrophil count/serum albumin

Data analysis was performed using SPSS 26.0 (IBM Inc., USA). The measurement data were expressed as mean ± standard deviation (SD), and the distributions of continuous variables were evaluated by Student’s t-test or the Mann-Whitney U test. Categorical variables were compared using the χ^2^ test and Fisher’s exact test. Neonatal birth weight was categorized as low birth weight (<2.5 kg), normal birth weight (2.5-4.0 kg), and macrosomia (>4.0 kg) (Xie et al., 2016). When RDS was regarded as the endpoint, receiver operating characteristic (ROC) curve analysis was used to determine the optimal cut-off values of SII, SIRI, and NAR. Logistic regression analysis was applied to assess the association between the related factors and RDS. Gender of neonates, adverse pregnancy and birth history of pregnant women, scarred uterus, amniotic fluid contamination, nuchal cord, placental abnormality, mode of delivery, birth weight were selected as covariates in the multivariate logistic regression analysis for the association between SII, SIRI, and NAR and NRDS, based on estimating the odds ratios (OR) and their 95% confidence intervals (CIs) in preterm infants with different gestational ages. *p*<0.05 was considered statistically significant.

## Results

### The clinical characteristics of premature infants

In this study, there were 1237 (57.2%) male neonates and 927 (42.8%) female neonates. There were 814 (37.6%) premature infants born to mothers with adverse pregnancy and birth histories before this pregnancy, and 1350 (62.4%) without. There were 235 (10.9%) premature infants born to mothers with scarred uteruses, and 1929 (89.1%) without. Amniotic fluid contamination, nuchal cord, and placental abnormality occurred in 38 (1.8%), 5 (0.2%), and 156 (7.2%) cases, respectively. There were 1738 (80.3%), 423 (19.5%), and 3 (0.1%) premature infants with birth weight <2.5 kg, 2.5-4.0 kg, and >4.0 kg, respectively. There were 1875 (86.6%), 100 (4.6%), and 28 (1.3%) premature infants with 1-minute Apgar scores of 7-10, 4-6, and <4 at birth, respectively. There were 1972 (91.1%), 32 (1.5%), and 2 (0.1%) premature infants with 5-minute Apgar scores of 7-10, 4-6, and <4, respectively (**Table 1**).

**Table 1:** Detailed clinical characteristics of premature infants, including birth history, maternal factors, and apgar scores.

Variables	Neonates (n=2164)
Gender of neonates	
Male, n (%)	1237 (57.2%)
Female, n (%)	927 (42.8%)
Adverse pregnancy and birth history of pregnant women	
No, n (%)	1350 (62.4%)
Yes, n (%)	814 (37.6%)
Scarred uterus	
No, n (%)	1929 (89.1%)
Yes, n (%)	235 (10.9%)
Perinatal conditions	
Amniotic fluid contamination	
No, n (%)	2126 (98.2%)
Yes, n (%)	38 (1.8%)
Nuchal cord	
No, n (%)	2159 (99.8%)
Yes, n (%)	5 (0.2%)
Placenta abnormality	
No, n (%)	2008 (92.8%)
Yes, n (%)	156 (7.2%)
Mode of delivery	
Spontaneous, n (%)	946 (43.7%)
Cesarean section, n (%)	1218 (56.3%)
Birth weight (kg)	
<2.5, n (%)	1738 (80.3%)
2.5-4.0, n (%)	423 (19.5%)
>4.0, n (%)	3 (0.1%)
1-minute Apgar score	
7-10, n (%)	1875 (86.6%)
4-6, n (%)	100 (4.6%)
<4, n (%)	28 (1.3%)
Unknown	161 (7.4%)
5-minute Apgar score	
7-10, n (%)	1972 (91.1%)
4-6, n (%)	32 (1.5%)
<4, n (%)	2 (0.1%)
Unknown	158 (7.3%)
Respiratory distress	
No, n (%)	1202 (55.5%)
Yes, n (%)	962 (44.5%)
Indexes of immune-nutritional status	
SII, median (P25, P75)	331.65 (206.27, 531.12)
SIRI, median (P25, P75)	1.20 (0.65, 2.29)
NAR, median (P25, P75)	0.16 (0.11, 0.22)

SII, systemic immune-inflammatory index; SIRI, systemic inflammatory response index; NAR, neutrophil-to-albumin ratio; p25, 25th percentile; p75, 75th percentile.

### Comparison of clinical characteristics among premature infants with or without RDS

There were 962 (44.5%) RDS and 1202 (55.5%) non-RDS neonates. The proportion of mothers had adverse pregnancy and birth history (40.6% vs. 35.2%, *p*=0.010), placenta abnormality (8.8% vs. 5.9%, *p*=0.009) in the RDS group were higher than those in the non-RDS group, while the proportion of scarred uterus was lower than that in the non-RDS group (8.9% vs. 12.4%, *p*=0.010). The proportion of birth weight <2.5kg, 1-minute Apgar score <7, and 5-minute Apgar score <7 in the RDS group was higher than that in the non-RDS group, respectively (all *p*<0.05). The levels of SII (363.97 (214.68, 621.30) vs. 308.84 (202.60, 459.60)), SIRI (1.33 (0.67, 2.95) vs. 1.13 (0.64, 1.93)), and NAR (0.17 (0.12, 0.25) vs. 0.15 (0.10, 0.20)) in premature infants with RDS was significantly higher than those in premature infants without RDS (all *p*<0.05) (**Table 2**).

**Table 2:** Comparison of clinical characteristics and inflammatory markers among premature infants with and without Respiratory Distress Syndrome (RDS).

Variables	Non-RDS group (n=1202)	RDS group (n=962)	*p* values
Gender of neonates			
Male, n (%)	687 (57.2%)	550 (57.2%)	1.000
Female, n (%)	515 (42.8%)	412 (42.8%)	
Adverse pregnancy and birth history of pregnant women			
No, n (%)	779 (64.8%)	571 (59.4%)	0.010
Yes, n (%)	423 (35.2%)	391 (40.6%)	
Scarred uterus			
No, n (%)	1053 (87.6%)	876 (91.1%)	0.010
Yes, n (%)	149 (12.4%)	86 (8.9%)	
Perinatal conditions			
Amniotic fluid contamination			
No, n (%)	1179 (98.1%)	947 (98.4%)	0.622
Yes, n (%)	23 (1.9%)	15 (1.6%)	
Nuchal cord			
No, n (%)	1198 (99.7%)	961 (99.9%)	0.390
Yes, n (%)	4 (0.3%)	1 (0.1%)	
Placenta abnormality			
No, n (%)	1131 (94.1%)	877 (91.2%)	0.009
Yes, n (%)	71 (5.9%)	85 (8.8%)	
Mode of delivery			
Spontaneous, n (%)	508 (42.3%)	438 (45.5%)	0.138
Cesarean section, n (%)	694 (57.7%)	524 (54.5%)	
Birth weight (kg)			
<2.5, n (%)	873 (72.6%)	865 (89.9%)	<0.001
2.5-4.0, n (%)	327 (27.2%)	96 (10.0%)	
>4.0, n (%)	2 (0.2%)	1 (0.1%)	
1-minute Apgar score			
7-10, n (%)	1036 (86.2%)	839 (87.2%)	<0.001
4-6, n (%)	34 (2.8%)	66 (6.9%)	
<4, n (%)	7 (0.6%)	21 (2.2%)	
5-minute Apgar score			
7-10, n (%)	1063 (88.4%)	909 (94.5%)	0.008
4-6, n (%)	10 (0.8%)	22 (2.3%)	
<4, n (%)	0 (0)	2 (0.2%)	
Indexes of immune-nutritional status			
SII, median (P25, P75)	308.84 (202.60, 459.60)	363.97 (214.68, 621.30)	<0.001
SIRI, median (P25, P75)	1.13 (0.64, 1.93)	1.33 (0.67, 2.95)	<0.001
NAR, median (P25, P75)	0.15 (0.10, 0.20)	0.17 (0.12, 0.25)	<0.001

RDS, respiratory distress syndrome; SII, systemic immune-inflammatory index; SIRI, systemic inflammatory response index; NAR, neutrophil-to-albumin ratio; p25, 25th percentile; p75, 75th percentile.

### ROC curve analysis of SII, SIRI, and NAR for RDS

**Figure 1:**
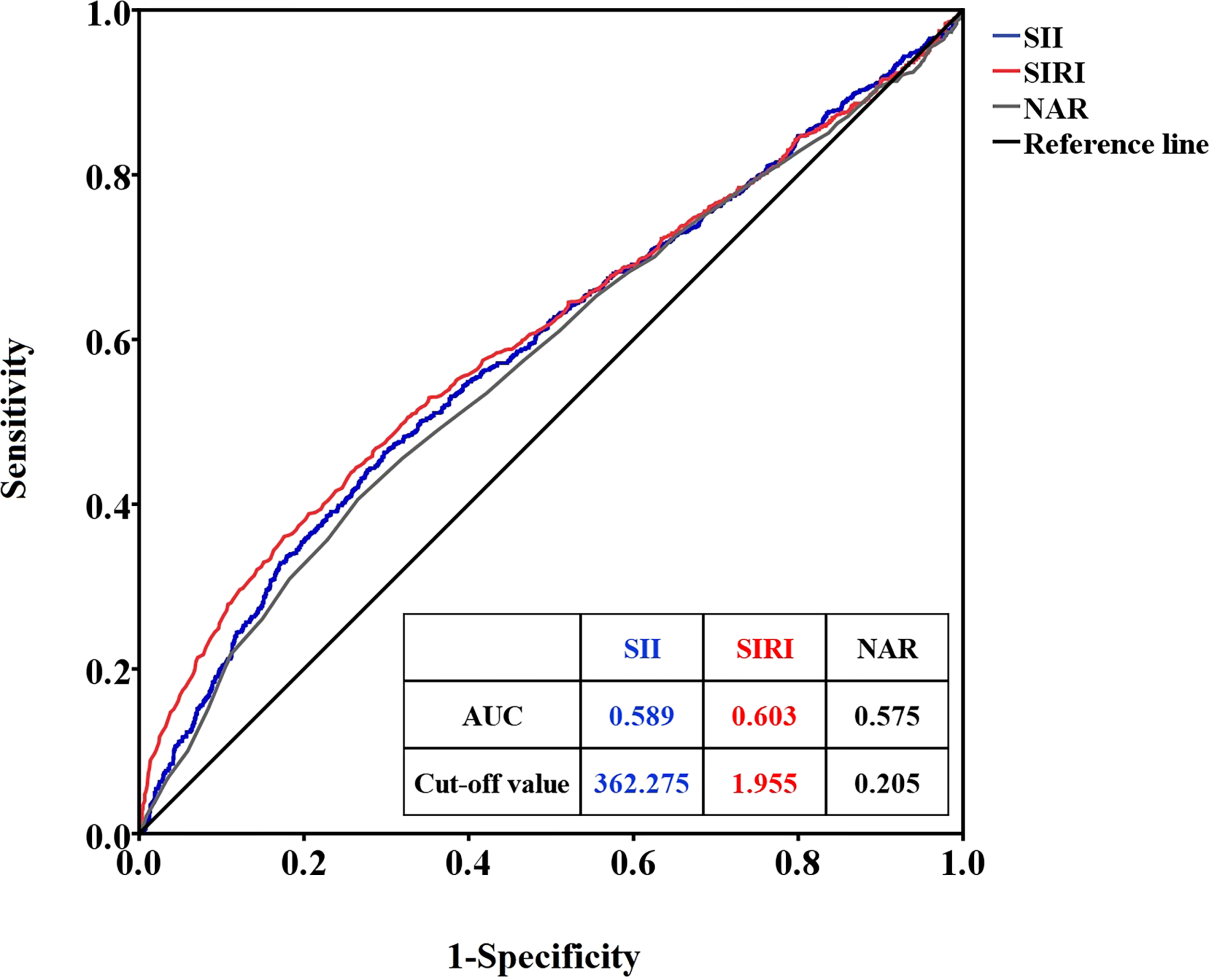
ROC curves of SII, SIRI, and NAR for RDS. SII, systemic immune inflammation index; SIRI, system inflammation response index; NAR, neutrophil-to-albumin ratio; RDS, respiratory distress syndrome.

ROC curve analysis was used to determine the optimal cutoff values of SII, SIRI, and NAR to distinguish premature infants with or without RDS. When RDS was considered as the endpoint of SII, SIRI, and NAR, the critical value of SII was 362.275 (sensitivity 50.6%, specificity 60.5%, area under the ROC curve (AUC)=0.589), the SIRI cutoff value was 1.955 (sensitivity 36.4%, specificity 75.5%, AUC=0.603), and the NAR cutoff value was 0.205 (sensitivity 36.5%, specificity 76.0%, AUC=0.575) (**Figure 1**).

### Logistic regression analysis of predictor factors of neonatal RDS

The results of the univariate analysis showed that adverse pregnancy and birth history of pregnant women (yes vs. no, odds ratio (OR): 1.261, 95% confidence interval (CI): 1.059-1.502, *p*=0.009), placenta abnormality (yes vs. no, OR: 1.544, 95% CI: 1.113-2.142, *p*=0.009), birth weight <2.5kg (<2.5kg vs. 2.5-4.0 kg, OR: 3.375, 95% CI: 2.639-4.317, *p*<0.001), SII high level (≥362.275 vs. <362.275, OR: 1.569, 95% CI: 1.322-1.862, *p*<0.001), SIRI high level (≥1.955 vs. <1.955, OR: 1.758, 95% CI: 1.460-2.117, *p*<0.001), and NAR high level (≥0.205 vs. <0.205, OR: 1.815, 95% CI: 1.506-2.187, *p*<0.001) were significantly associated with RDS in premature infants. Multivariate regression logistic analysis showed that adverse pregnancy and birth history of pregnant women (OR: 1.390, 95% CI: 1.154-1.675, *p*=0.001), placenta abnormality (yes vs. no, OR: 2.499, 95% CI: 1.732-3.607, *p*<0.001), birth weight <2.5kg (OR: 4.165, 95% CI: 3.203-5.414, *p<*0.001), SIRI high level (≥1.955 vs. <1.955, OR: 1.338, 95% CI: 1.021-1.753, *p*=0.035), and NAR high level (≥0.205 vs. <0.205, OR: 1.639, 95% CI: 1.270-2.115, *p*<0.001) were independent predictive factors for RDS in premature infants (**Table 3**).

**Table 3:** Logistic regression analysis of predictor factors of neonatal RDS.

Variables	Univariate		Multivariate	
	OR (95% CI)	*p* values	OR (95% CI)	*p* values
Gender of neonates (Male vs. Female)	1.001 (0.843-1.188)	0.993	1.172 (0.976-1.406)	0.089
Adverse pregnancy and birth history of pregnant women (Yes vs. No)	1.261 (1.059-1.502)	0.009	1.390 (1.154-1.675)	0.001
Scarred uterus (Yes vs. No)	0.694 (0.524-0.918)	0.011	0.832 (0.613-1.129)	0.238
Amniotic fluid contamination (Yes vs. No)	0.812 (0.421-1.565)	0.534	0.809 (0.403-1.623)	0.550
Nuchal cord (Yes vs. No)	0.312 (0.035-2.793)	0.297	0.431 (0.047-3.936)	0.456
Placenta abnormality (Yes vs. No)	1.544 (1.113-2.142)	0.009	2.499 (1.732-3.607)	<0.001
Mode of delivery (Cesarean section vs. Spontaneous)	0.876 (0.738-1.039)	0.128	0.915 (0.756-1.108)	0.364
Birth weight (kg)				
2.5-4.0	1.000 (reference)		1.000 (reference)	
<2.5	3.375 (2.639-4.317)	<0.001	4.165 (3.203-5.414)	<0.001
>4.0	1.703 (0.153-18.986)	0.665	1.885 (0.166-21.411)	0.609
SII (≥362.275 vs. <362.275)	1.569 (1.322-1.862)	<0.001	1.221 (0.976-1.527)	0.081
SIRI (≥1.955 vs. <1.955)	1.758 (1.460-2.117)	<0.001	1.338 (1.021-1.753)	0.035
NAR (≥0.205 vs. <0.205)	1.815 (1.506-2.187)	<0.001	1.639 (1.270-2.115)	<0.001

OR, odds ratio; CI, confidence interval; RDS, respiratory distress syndrome; SII, systemic immune-inflammatory index; SIRI, systemic inflammatory response index; NAR, neutrophil-to-albumin ratio.

### Analysis of secondary respiratory infection risk in preterm infants with and without RDS

We also evaluated the effect of RDS on the risk of secondary respiratory infections in preterm infants, showing that the proportion of RDS preterm infants who developed secondary infections was significantly higher than in the non-RDS group. Specifically, the rate of pneumonia was higher in the RDS group than in the non-RDS group, and the rate of sepsis was higher in the non-RDS group than in the RDS group. Multivariate logistic regression analysis showed that immunoinflammatory markers and RDS are associated with secondary respiratory infection in preterm infants after adjusting for other relevant factors (such as gender of neonates, adverse pregnancy and birth history of pregnant women, scarred uterus, amniotic fluid contamination, nuchal cord, placenta abnormality, mode of delivery, birth weight). The specific data and results of this section will be presented in another of our studies.

## Discussion

The pathogenesis of neonatal RDS is a diffuse lung injury process in which irregular inflammatory substances exudate from alveolar cells in multiple lung fields may occur on both sides of the lungs (Fernando et al., 2021). RDS is a common critical disease in premature infants, and the prognosis of premature infants with RDS is often worse than that of children and adults due to immature organ and tissue development, immune function, susceptibility to bronchopulmonary dysplasia, lung biology, low maturity and other reasons (Gillies et al., 2012; Stading et al., 2021). SII, SIRI, and NAR are important indicators of systemic inflammation and immunity, and elevated levels of these indicators can reflect lung tissue inflammation in children with RDS.

The research on peripheral blood lymphocytes, neutrophils, and platelets is increasing, and the detection of these indicators is non-invasive and rapid. Neutrophils can activate adaptive immunity (Kolaczkowska and Kubes, 2013). Lymphocytes are the main cells of the adaptive immune system, and both play an important role in inflammatory response (Wang and Zhou, 2020). Platelets directly recognize, isolate, and kill pathogens, and activate and recruit white blood cells to sites of infection and inflammation (Trugilho et al., 2017). Platelets can also indirectly participate in innate and adaptive immunity, and enhance the ability of immune cells to phagocytose and kill pathogens, further leading to inflammation of blood vessels and injured tissues (Nicolai and Massberg, 2020). Nupponen I et al. found that postnatal neutrophil activation is indicative of systemic inflammation and associated with RDS in preterm infants (Nupponen et al., 2002). Chen et al. found that the platelet, neutrophil, and monocyte count were significantly higher in infants with moderate-severe bronchopulmonary dysplasia (BPD) compared to infants without BPD (Chen et al., 2019b). Plateletcrit level was significantly increased in infants with respiratory distress than controls (Mishra et al., 2021). In premature infants with RDS, significant activation of circulating phagocytes occurs after the initiation of mechanical ventilation, independent of surfactant administration (Turunen et al., 2006). High serum ischemia-modified albumin level may be a valuable indicator of hypoxia in infants with RDS (Kahveci et al., 2016). SII, SIRI, and NAR are comprehensive indicators of these haematological parameters, which can comprehensively reflect the level of these indicators. Levels of SII, SIRI, and NAR may be associated with the risk of RDS in preterm infants. Ufuk Cakir et al. demonstrated that a higher SII level was a predictor for RDS among premature infants (Cakir et al., 2024). There is no reported relationship between SIRI, NAR and neonatal RDS. The purpose of this study was to analyze the predictive value of SII, SIRI and NAR in RDS risk, and results showed that high SIRI and NAR levels were independently associated with RDS in premature infants.

There have been some reports on the relationship between SIRI, NAR and some respiratory diseases. SIRI was associated with an increased risk of all-cause death in patients with asthma (Ke et al., 2023). Zhao et al. found that SIRI was a potential predictor of severe pneumonia in patients with cerebral hemorrhage (Zhao et al., 2023). SIRI was an independent prognostic factor in patients with lung cancer (Zuo et al., 2023; Li et al., 2019; Wang and Li, 2024). Gao et al. revealed that SIRI can predict the severity and prognosis of pulmonary arterial hypertension (Gao et al., 2024). And SIRI also associated with idiopathic pulmonary fibrosis (Zinellu and Paliogiannis, 2020), pneumonia in children (Elmeazawy et al., 2024), and severity of coronavirus disease 2019 (COVID-19) (Gutiérrez-Pérez et al., 2022). In addition, NAR was associated with the stage of non-small cell lung cancer (Varim et al., 2022), mortality in COVID-19 infected patients (Varim and Yaylaci, 2020), and prognosis of pulmonary embolism (Lian et al., 2024).

Moreover, placenta abnormality was a risk factor for RDS in premature infants in this study. Placenta accreta spectrum (PAS) neonates have higher rates of respiratory morbidity (Munoz et al., 2022). Infants born to mothers with placenta previa had a higher risk for RDS (Lin et al., 2001; Bekku et al., 2000; Lal and Hibbard, 2015). Placenta previa is a risk factor for neonatal transient tachypnea (Tsuda et al., 2014). Overall, placental abnormality is a risk factor for RDS in newborns. Moreover, low birth weight was the main risk factor for RDS (Condò et al., 2017; Hu et al., 2024b). Low birth weight was a risk factor for RDS in premature infants in this study. However other studies suggest that the risk of RDS does not seem to be related to the birth weight of preterm infants (Chard et al., 1997). In addition, Fang et al. demonstrated that male neonates had a higher risk of RDS than females (Fang et al., 2024). J S Anadkat found that the male sex independently increases the risk for RDS in preterm infants (Anadkat et al., 2012). However, in terms of gender, this study did not get the same results. Some studies suggested that cesarean section was the risk of RDS (Condò et al., 2017; Indraccolo et al., 2019; Wondie and Legesse, 2023), but this study did not get similar results. Scarred uterus, amniotic fluid contamination, and nuchal cord were not associated with RDS in this study, and these need to be confirmed by more researches.

High SIRI (≥1.955) and NAR level (≥0.205), adverse pregnancy and birth history of pregnant women, placenta abnormality, birth weight <2.5kg were independent predictive factors for RDS in premature infants. The study has some limitations that are worth noting. First, as a retrospective study, other possible influencing factors of RDS were not included in the analysis, so the results may be biased. Second, this study only collected and analyzed the data of the first laboratory examination when premature infants were admitted to hospital, and failed to select inflammatory indicators at different time points for dynamic analysis. And the assessment results for these factors and RDS may have been biased. Third, this study is a single-centre study and lacks external data for verification. In the future, further expansion of clinical sample sizes and multi-centre, prospective studies are needed to confirm the value of SII, SIRI, and NAR in assessing the risk of RDS.

Early identification and early intervention of risk factors for RDS are very important for disease prevention and control. The occurrence of RDS is influenced by several factors during pregnancy and perinatal period. In the future research, there are several aspects worthy of attention. First, there are different risk factors for the development of RDS. What is the mechanism of action of these risk factors in the formation of alveolar surfactant and lung development and maturation? Second, there are a large number of risk factors for RDS, and controlling which risk factors can significantly reduce the incidence of RDS and improve the prognosis.

## Conclusions

We identified several independent predictive factors for RDS in premature infants, including high SIRI and NAR levels, adverse pregnancy and birth history, placental abnormality, and birth weight <2.5 kg. These findings emphasize the importance of monitoring these parameters in the early assessment of RDS risk among preterm neonates. This study is among the first to demonstrate the predictive value of high SIRI and NAR levels for RDS in this population. Additionally, placental abnormalities and low birth weight were significant risk factors, reinforcing the role of maternal and fetal factors in the development of RDS. In other words, premature infants with placental abnormalities, low birth weight, and high SIRI and NAR levels should be alert to the risk of RDS.

## Data Availability

The original contributions presented in the study are included in the article/supplementary material. Further inquiries can be directed to the corresponding author.
